# Long-term Remission in Functioning Pituitary Adenomas after Medical Therapy Withdrawal: A Chance for Cushing’s Disease

**DOI:** 10.2174/0118715303328077240719055819

**Published:** 2024-07-30

**Authors:** Alessandro Mondin, Filippo Ceccato, Carla Scaroni, Mattia Barbot

**Affiliations:** 1 Department of Medicine-DIMED, University of Padova, Padova, Italy;; 2 Endocrinology Unit, University Hospital of Padova, Padova, Italy

**Keywords:** Cushing’s disease, pasireotide LAR, medical therapy, CD remission, somatostatin analogues, pituitary adenomas

## Abstract

**Background:**

The possibility of sustained disease remission in functioning pituitary adenomas after drug withdrawal is well-known for prolactinomas and it has also been described in a subset of acromegalic patients. On the contrary, medical treatment for Cushing’s Disease (CD) is generally considered a life-long measure except for previously radio-treated patients. Sparse evidence of spontaneous remissions in CD has been reported, mainly related to a possible pituitary tumor apoplexy. To the best of our knowledge, none of these cases has included the use of a pituitary targeting agent.

**Case Presentation:**

Herein, we have reported the case of a radiotherapy-naïve patient with persistent CD after pituitary surgery who participated in the CSOMG230 trials, presenting sustained remission after Long-acting Release (LAR) pasireotide withdrawal. Under monthly pasireotide LAR 40 mg, the patient achieved urinary hormone control and clinical signs of cortisol excess normalization. After 8 years of treatment, the patient completed the study protocol and had to withdraw the drug as it was no longer available for CD in Italy. Before starting new therapies, we reassessed hormone levels that were surprisingly within normal ranges. At 24 months after the last dose of pasireotide, the patient was still in clinical and biochemical full remission. We have also briefly reviewed previous cases of sustained remission after somatostatin analogues withdrawal in other functioning pituitary adenomas.

**Conclusion:**

Far from the general rule, this case suggests that prolonged treatment with pasireotide LAR might induce a durable CD remission. A dose down-titration/suspension might be considered in patients well-controlled on long-term therapy and with negative pituitary imaging. However, close monitoring is recommended given the high rate of complications in untreated patients.

## 1 INTRODUCTION

Cushing’s Disease (CD) is a rare condition, characterized by increased morbidity and mortality, especially if not timely managed [1]. Transsphenoidal Surgery (TSS) is the first-line option in CD with a remission rate ranging between 80 and 90% of cases. The overall outcome of surgery is further weakened by postoperative recurrences in approximately 20% of initially “cured” patients. In all these cases, other options should be considered to control cortisol excess [2]. Among second-tier options, medical therapy has gained increasing interest in the last decade owing to the availability of new compounds.

In other functioning pituitary adenomas, the possibility of a medically-induced disease remission has been reported [3, 4]; although a long-term control with cortisol-lowering medications is possible in CD, treatment discontinuation has been only anecdotally observed in radiotherapy-naïve patients. These apparent spontaneous remissions have mainly occurred while on steroidogenesis inhibitors and have been probably related to silent tumour infarction [5]. Indeed, most of the available drugs for CD do not target the pituitary adenoma directly but aim to reduce cortisol overproduction by inhibiting adrenal steroidogenesis. Conversely, cabergoline and Somatostatin Analogues (SSAs) directly target the pituitary adenoma. In particular, the multiligand SSA pasireotide acts on four out of five Somatostatin Receptor (SSTR) isoforms, with a particularly high affinity for the SSTR5, which is the most prevalent isoform in corticotroph adenomas. Glucocorticoid excess indeed downregulates SSTR2 expression, explaining the efficacy of pasireotide in treating CD compared to first-generation SSAs [6]. Apart from cortisol control, pasireotide has proven to reduce tumour volume in both ACTH and GH-secreting adenomas [7].

To our knowledge, no prior report has described disease remission after the discontinuation of a pituitary-acting drug in a CD patient who did not receive prior pituitary irradiation. Herein, we have reported the case of a patient with persistent CD who was enrolled in the CSOMG230 trials and experienced sustained remission after Long-acting Release (LAR) pasireotide withdrawal due to study termination.

We have also reviewed all cases of remission after medical treatment discontinuation in radiotherapy naïve patients with CD or acromegaly.

## 2 BACKGROUND

Regarding our clinical case, all the presented data were retrieved from the hospital charts. The CSOM230G trial was approved by the Ethics Committee of Padua and the patient provided signed informed consent to participate in the core study and in its extension phases; afterward, the patient provided informed consent to publish the results in the form of a clinical case.

The phase III trial (CSOM230G2304) consisted of a 12- month core phase [8], followed by an open-label extension phase [9], for the evaluation of the efficacy and safety of intramuscular pasireotide LAR in CD. After 24 months of treatment, the patient entered the optional phase IV extension period (CSOM230B2412). During the study, participants’ biochemical and hormonal analyses were centralized as per study protocol to Quintiles, Marietta, GA, USA, and Q2 Solutions Co. Ltd, Beijing, China. Ultra-performance Liquid Chromatography-tandem Mass Spectrometry (LC-MS/MS) (Waters Corp., Milford, MA, USA) was used to determine morning serum cortisol, Late-night Salivary Cortisol (LNSC), and Urinary Free Cortisol (UFC; normal range 15.9-166.5 nmol/24 h) levels. Plasma Adrenocorticotropic Hormone (ACTH) levels were analyzed using a radioimmunoassay (Immulite 2000 ACTH PIL2KAC-15, Siemens Medical Solutions Diagnostics, Los Angeles, CA, USA). Efficacy analysis was based on the mean of three UFC samples at each time point. Pituitary Magnetic Resonance Imaging (MRI) was performed at the study site and images were collected and analyzed by a single independent blinded radiologist to assess tumour volume changes every six months.

At diagnosis, UFC was measured at our center by radioimmunoassay (Biodata Diagnostic, Rome, Italy; normal range 90-694 nmol/24 h); since December 2011, our laboratory switched to an LC-MS/MS assay for UFC (Agilent Technologies, Palo Alto, CA, USA; normal range 16-168 nmol/24 h). LNSC was measured with a radioimmunoassay kit (Radim, Rome, Italy) until 2014, and thereafter with an LC-MS/MS assay (Agilent Technologies, Palo Alto, USA). Plasma ACTH was measured with an immunoassay (Immulite 2000, Siemens Healthcare, Erlangen, Germany). At the last follow-up, dexamethasone levels (following 1 mg of overnight dexamethasone suppression) were measured by LC-MS/MS assay, as previously described [10].

As far as the literature review is concerned, the authors searched the PubMed database through combinations of relevant words (*i.e*., remission AND functioning adenoma type AND drug name) and further expanded their search through the analysis of relevant citations of the identified publications.

## 3 CLINICAL CASE PRESENTATION

A 54-year-old woman was referred to our unit for abnormal bruisability in the upper limbs. She also reported weight gain (5 kg in the last year, following menopause), hirsutism, rounding of the face, and rubeosis. She did not exhibit large purple striae or dorsal fat pad. She had a 2-year-long history of arterial hypertension (fairly controlled on a combination of angiotensin-converting enzyme inhibitor, calcium channel blocker, and thiazide diuretic), dyslipidaemia on statin treatment, osteopenia at the lumbar spine, a pulmonary node with a radiologically benign appearance, and a monoclonal gammopathy of undetermined significance on regular hematologic follow-up. She denied alcohol intake, the use of corticosteroids, or familial history of endocrine diseases. She presented altered first-line tests for endogenous hypercortisolism and unsuppressed ACTH levels, suggesting an ACTH-dependent Cushing’s Syndrome (CS). Although second-line tests pointed toward a pituitary source of ACTH secretion, further diagnostic procedures were required due to the negative finding at the pituitary MRI and the presence of a pulmonary nodule (Table **1**). Chromogranin A and neuron-specific enolase were within normal range and no significant uptake by the pulmonary lesion was observed at the Octreoscan. Bilateral inferior petrosal sinus sampling finally confirmed a central to peripheral ACTH gradient consistent with CD diagnosis, both at baseline and following human corticotropin-releasing hormone stimulation.

Therefore, the patient was subjected to pituitary surgery, but unfortunately, the early postoperative serum cortisol level was 447 nmol/l, proving ineffective TSS, and histological examination revealed normal pituitary tissue. In fact, the patient maintained an overt clinical picture and biochemical hypercortisolism (UFC 1.53-fold the ULN) was soon confirmed; thus, a combination of low-dose ketoconazole (200 mg daily) and cabergoline (2 mg weekly) was started. Due to uncontrolled hypercortisolism in the following months (UFC 2-fold above the ULN), the dosage of both drugs was progressively increased up to 400 mg daily and 3.5 mg weekly, respectively, without achieving full hormone control; further escalation of ketoconazole was not possible due to liver enzyme elevation. The pituitary MRI remained negative.

**Fig. (1):**
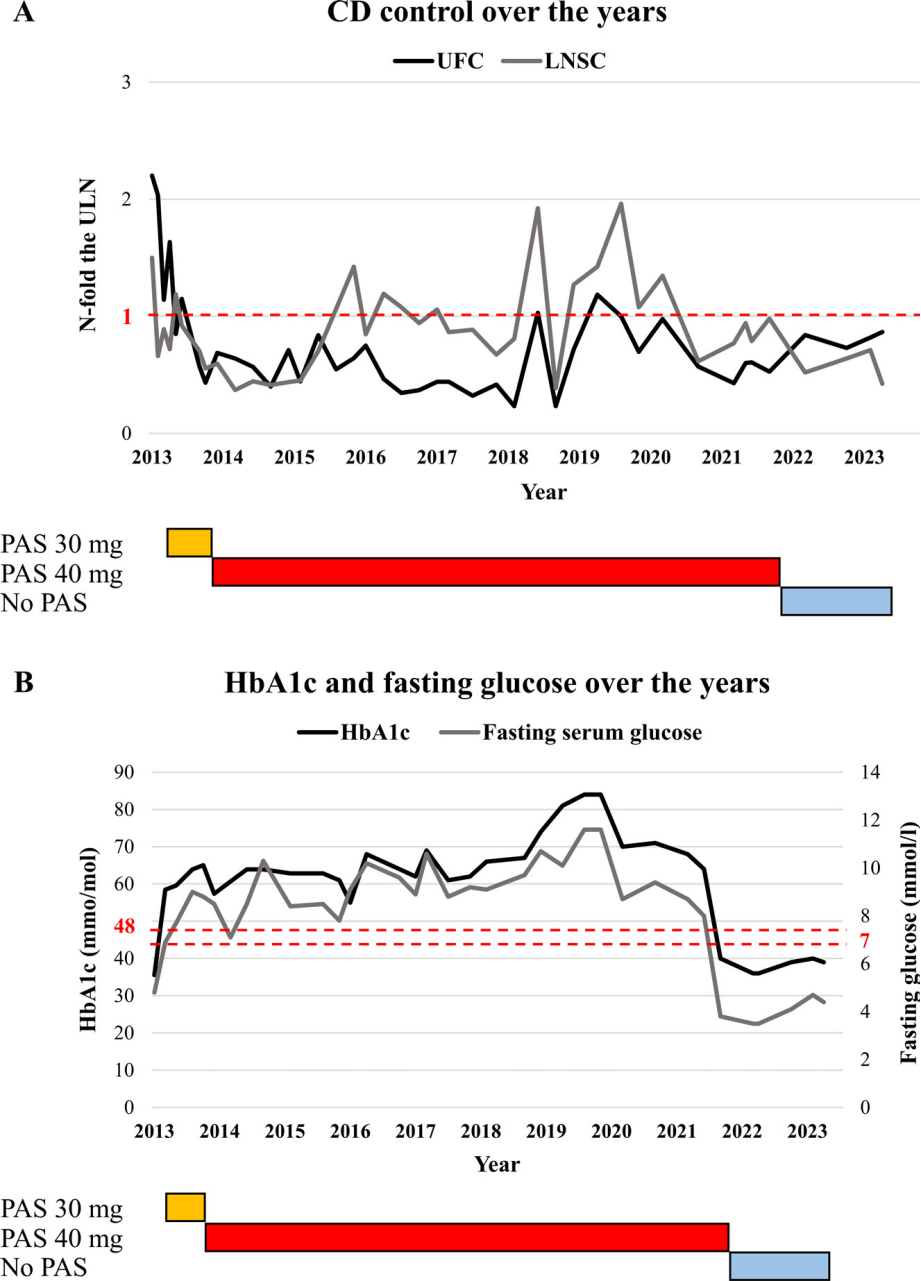
(**A**) Disease control over the years. (**B**) Glucose homoeostasis over the years. **Abbreviations:** CD, Cushing’s disease; UFC, urinary free cortisol; LNSC, late-night salivary cortisol; N, number; ULN, upper limit of normal; PAS, pasireotide long-acting release; HbA1c, glycosylated haemoglobin.

Since signs and symptoms of the disease were still present, other therapeutic options were discussed with the multidisciplinary group dedicated to pituitary diseases and the patient. Given the negative pituitary MRI, the probability of success of a second TSS was low and the patient refused to undergo bilateral adrenalectomy. Therefore, it was proposed that the patient be enrolled in the phase III CSOM230G2304 trial to assess the efficacy and safety of pasireotide LAR in refractory CD [8]. Three years after the surgery, in September 2013, the patient signed the informed consent form, and after the appropriate wash-out period, she was screened and found to meet the inclusion criteria (UFC 2.2-fold the ULN and LNSC 1.5-fold the ULN). She was randomly assigned to the PAS 30 mg i.m. arm every 4 weeks as per the study protocol. No adverse events were registered in the first three months of treatment, but due to partially controlled UFC (*i.e*., 1.63-fold the ULN) after 3 months of treatment, a dose escalation to once-monthly PAS LAR 40 mg i.m. was performed as per the protocol. Following this adjustment, the patient achieved optimal disease control with UFC normalization and circadian rhythm restoration (Fig. **1A**). After 4 months of treatment, the patient developed mild diabetes mellitus, and oral hypoglycaemic treatment with 1500 mg of metformin daily was started. An increase in anti-diabetic treatment (metformin 2200 mg and sitagliptin 100 mg daily) was required to obtain an acceptable control (glycosylated haemoglobin 57 mmol/mol).

Following the excellent response in the core phase of the study, the patient entered the extension protocol of CSOM230G2304 [9]. Despite persistently normal UFC and circadian rhythm, she presented a progressive worsening of glucose homeostasis requiring an association of metformin, DDP4 inhibitor, sodium-glucose cotransporter-2 inhibitor, gliclazide, and pioglitazone due to poor glycaemic control (Fig. **1B**). This multiple association was required since the patient did not tolerate weekly subcutaneous glucagon-like peptide-1 agonist and refused to undergo daily insulin treatment. The pituitary imaging remained negative throughout the follow-up.

Given the good control of cortisol secretion, the patient was maintained on pasireotide LAR until the study ended (last administration in January 2022). At that time, after more than 8 years of pasireotide use, the patient was asked to switch to a subcutaneous formulation of the same drug (since the LAR formulation has not been registered for CD treatment in Italy), but she refused it.

To reassess disease status, a short wash-out period of 2 months was arranged. As expected, we observed an improvement in glucose homeostasis, but surprisingly, there was neither clinical nor biochemical evidence of hypercortisolism relapse at the first re-evaluation. To rule out the possible effect of potential residual activity of the last pasireotide administration, the patient was maintained without cortisol-lowering medications and periodically retested at 6, 12, 18, and 24 months after the last injection. Hormone levels, assessed *via* UFC and LNSC, remained within normal range throughout the entire period of observation (Fig. **1A**), with only a borderline response of both ACTH and cortisol to the desmopressin challenge [11]. Twenty-four months after drug discontinuation, the patient had impaired cortisol suppression after a 1 mg overnight dexamethasone suppression test, but dexamethasone levels resulted in being under the lower limit of normal despite the patient reporting correct drug consumption. UFC and LNSC were, however, normal at the time. To exclude a possible mild recurrence, we performed a low-dose Liddle test [12]. This time, cortisol and dexamethasone levels resulted in being adequate, confirming remission (Table **2**). The screening for possible causes of malabsorption exhibited negative results. Meanwhile, following pasireotide discontinuation, there was a complete recovery of normal glucose homeostasis, with discontinuation of concomitant hypoglycaemic drugs; at the last available examination, the patient presented a normal glycosylated haemoglobin value (39 mmol/mol) without any treatment.

At 24 months after the last pasireotide dose, the patient still had normal cortisol levels without any ongoing cortisol-lowering treatment and no signs or symptoms of CD (Table **2**).

Of note, during PAS treatment, the patient did not report symptoms consistent with adrenal insufficiency and hormone level did not fall below the normal range (morning serum cortisol nadir 301 nmol/l; morning salivary cortisol nadir 4.1 nmol/l; ACTH nadir 11 ng/l). Fig. (**2**) summarizes the clinical case timeline.

## 4 DISCUSSION

Although rare, some cases of spontaneous CD remission have been previously described. As pinpointed by a recent systematic review [5], while postsurgical hypocortisolemia is a well-established criterion for remission, the definition of spontaneous CD remission is unclear. The authors suggest considering spontaneous CD remission in cases of amelioration/reversal of the clinical picture and normal hormonal findings (*i.e*., normal first-line tests for CS) in patients who are not taking medical treatment and have not received prior radical surgical treatments (either pituitary or adrenal) or pituitary irradiation. Following this definition, Popa Ilie and collaborators identified 23 cases of spontaneous disease remission. The mean time to remission was 5 months from diagnosis, and the reported follow-up varied from 6 to 130 months, with relapse occurring in 39% (9/23) of patients. Pituitary Tumour Apoplexy (PTA) was the most common causative event (91%) and was radiologically documented in 43% of patients. Notably, most CD patients harboured a microadenoma; it has been supposed that a PTA in this kind of lesion may easily infarct the entire tumour leading to spontaneous remission without causing significant clinical symptoms or other hormonal deficiencies, although the risk of hypocortisolism should always be considered [13]. Interestingly, 22% of patients were treated with steroidogenesis inhibitors, not directly targeting the pituitary (Table **3**) [5, 13-16]. In these patients, a rise in ACTH levels is often registered following the loss of feedback from controlled peripheral hypercortisolism. Therefore, the authors of the review [5] speculated that an increase in the metabolic needs of the adenomatous cells, exceeding their blood supply, could result in acute ischemia, followed by spontaneous infarction of the corticotroph adenoma (*i.e*., resolution of CD). Regarding ketoconazole, especially in the absence of a rise in ACTH levels, other mechanisms have been proposed, including a direct effect of the drug on corticotroph cells. This latter hypothesis is supported by *in vitro* evidence, but *in vivo* data are less clear [16, 17]. Dickstein and colleagues performed a follow-up of their previously described spontaneous remissions [13], and a patient previously treated with ketoconazole, despite the lack of clinical or biochemical relapse, exhibited pituitary tumour growth. Histologic evaluation revealed positive ACTH staining, hinting at a remission based on the phenotypic switch to a silent corticotroph adenoma [14]. The hypothesis of a cyclical CS was unlikely in these patients, based on the length of biochemical remission and the reversal of the clinical picture, although this eventuality was described in some cases of cyclic CS with long hypo- or normo-cortisolemic secretion phases [18].

Returning to our patient, as she has been regularly followed for nearly 15 years and has not shown any intermittency in hormone secretion, the hypothesis of a cyclic CS was extremely unlikely. The occurrence of asymptomatic PTA or the switch to a silent phenotype were remote events as well, although not fully switchable. Notably, in our case, there was a pivotal difference from prior reports on spontaneous remission following the withdrawal of steroidogenesis inhibitor, *i.e*., the use of a pituitary targeting agent likely responsible for CD remission. Indeed, the anti-tumoural activity of this drug has been well documented both *in vitro* and *in vivo*. Pasireotide was found to reduce cell proliferation (ranging from 10 to 70%) in primary cultures from six corticotroph adenomas [19] and significantly inhibit proopiomelanocortin transcription and cell proliferation in AtT20 cells. This latter finding could not be reproduced in SSTR-5 knockout cells, suggesting an important role of this receptor in the anti-tumoural effect that may involve a reduction in AKT phosphorylation. Moreover, pasireotide reduced tumour weight in a mice AtT20-xenograft model compared to untreated controls, with an *in vivo* reduction of pituitary tumour transforming gene messenger ribonucleic acid [20]. A likely additional indirect anti-tumoural mechanism was elucidated in primary cultures from non-functioning pituitary adenomas, where pasireotide impaired cell viability *via* vascular endothelial growth factor suppression [21]. Regarding *in vivo* data, an interesting post-hoc analysis of the PAPE study described an increase in T2-weighted MRI signals in treated somatotroph adenomas; patients with these radiological findings exhibited a more pronounced decrease in IGF-1 irrespective of tumour shrinkage. Hence, it may be speculated that this signal alteration is a consequence of cell degeneration and/or tumour cell necrosis despite unaltered tumour volume [22]. Finally, pasireotide can induce significant tumour shrinkage in CD [7] and has previously led to disease remission in some cases of somatotroph adenoma [23-25].

To the best of our knowledge, this is the first report of a CD remission following pituitary-targeting medical treatment in a radiotherapy naïve patient. Cortisol-lowering medications are generally considered a life-long option for CD patients, especially because most of the treatments do not specifically target the corticotroph tumour. Conversely, in our case treated for more than 8 years with pasireotide, the absence of clinical symptoms and the complete normalization of all first-step diagnostic tests for diagnosing cortisol excess are strong criteria to label our patient as in remission after treatment discontinuation. Notably, our patient had a negative pituitary MRI, suggesting the presence of a tiny microadenoma that might be more sensitive to the antitumoral activity of pasireotide compared to larger lesions. The modest ACTH and cortisol response to desmopressin (present at the same level in baseline) might hint at a future of disease relapse [11], but 24 months following drug withdrawal, the patient still does not show signs or symptoms of disease and has no hormone impairment. Our experience suggests the possibility of down-titrating/suspending medical treatment in selected patients with well-controlled disease on long-term pasireotide LAR therapy with negative pituitary MRI; nevertheless, given the scarce clinical experience in this scenario and the high rate of complications of untreated CD, a close clinical and biochemical monitoring remains mandatory.

Although a novelty in CD, medically induced remission is rather common in other functioning pituitary adenomas.

In most cases of prolactinomas, Dopamine Agonist (DA) cabergoline is the front-line agent because it reduces both hormone levels and tumour volume [26]. Remission after a long-term course of DA has been extensively described for prolactin-secreting adenoma. Indeed, although DAs can be considered for long-term treatment, a subgroup of patients may achieve persistent normoprolactinemia even after drug discontinuation; a few months after withdrawal, a rebound of prolactin levels is nevertheless possible. Current guidelines suggest that DAs withdrawal can be considered after at least two years of treatment in patients who achieve normoprolactinemia and tumour disappearance [3]. The rate of long-term normoprolactinemia after DAs withdrawal is highly variable in literature, as recently pinpointed by a metanalysis on the topic (range 0-75%) [27]; several factors have been linked to sustained remission, including older age, longer duration of medical treatment, normoprolactinemia on low dose DAs prior to discontinuation, cabergoline over bromocriptine treatment, the presence of a micro- over a macroadenoma at baseline, and significant shrinkage/disappearance of the tumour prior to withdrawal [27, 28]. Interestingly, Zou and collaborators reported that actual recurrence (*i.e*., tumour enlargement or recurrence irrespective of prolactin levels) is rare after drug suspension, suggesting that isolated relapsing hyperprolactinemia may not need treatment; further studies are needed to better address this topic [29].

Although rare, long-standing remission after medical treatment discontinuation in radiotherapy-naïve acromegalic patients has been described as well. First-generation SSAs are of choice for acromegalic patients when surgery is not feasible or has proven to be ineffective [30]. Although medical treatment with SSAs in radiotherapy-naïve patients is usually considered a long-life measure, the possibility of its withdrawal has been explored in some clinical cases and study series (Table **4**) [4, 23-25, 31-41]. Several reports on first-generation SSAs have shown that a subset of well-controlled patients may reach remission after drug discontinuation. Notably, Sala and collaborators extended the period of observation of their previous work [41] up to 10 years, confirming long-term remission in approximately 20% of their cohort [4]. Lower Insulin Growth Factor 1 (IGF-1) levels during treatment were identified as predictors of sustained remission after SSA discontinuation [4]. Similarly, pasireotide was also observed to induce sustained remission after withdrawal in a similar setting [23-25]. Interestingly, two cases of long-term remission after pegvisomant withdrawal were described, suggesting a possible unknown anti-tumoural effect of this drug [40]. Sparse evidence supporting cabergoline withdrawal in acromegaly is also available [32, 39].

## CONCLUSION

The case presented in this work suggests that prolonged treatment with pasireotide LAR might induce a durable CD remission. As is the case for other functioning pituitary adenomas, a dose down-titration/suspension might be considered in patients well-controlled on long-term pasireotide LAR therapy and with negative pituitary MRI. However, close monitoring is recommended given the high rate of complications in untreated patients.

## AUTHORS’ CONTRIBUTIONS

A.M. and M.B. wrote the manuscript text; A.M. prepared figures; F.C. and M.B. followed the patient throughout the years; C.S. supervision; all authors reviewed the final version of the manuscript.

## Figures and Tables

**Fig. (2):**
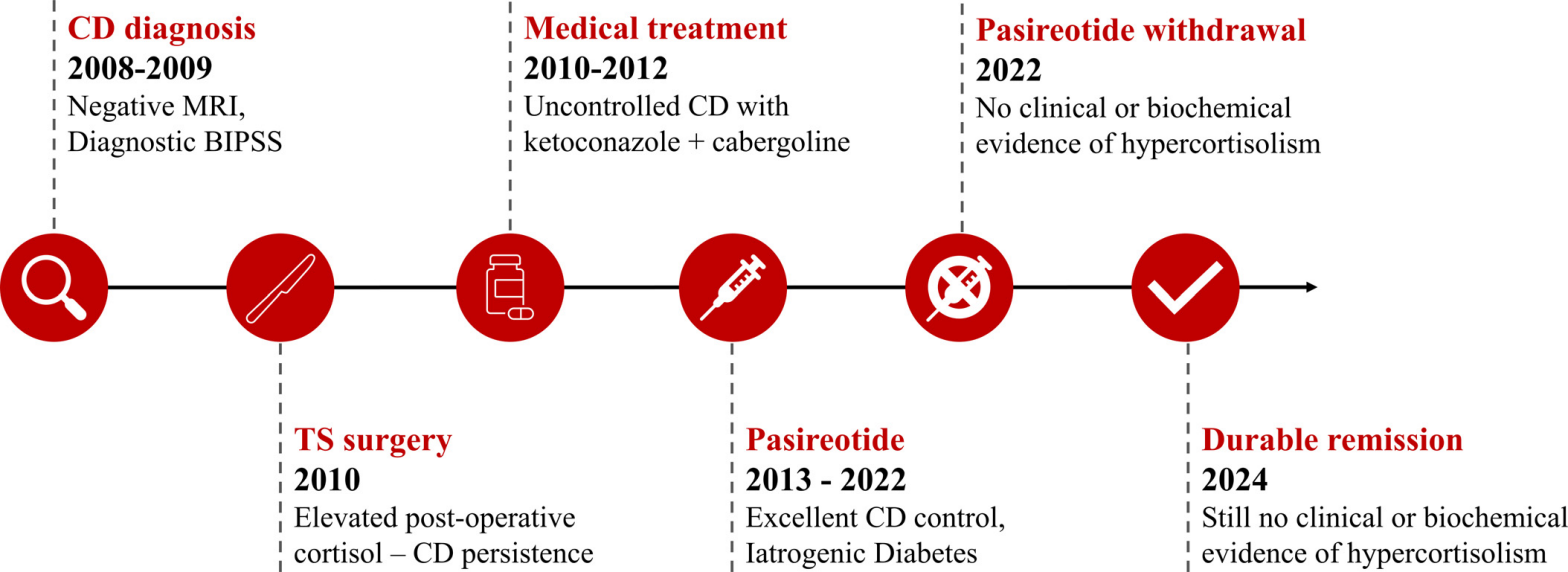
Clinical case timeline. **Abbreviations:** CD, Cushing’s Disease; MRI, magnetic resonance imaging; BIPSS, bilateral inferior petrous sinus sampling, TS, trans-sphenoid.

**Table 1:** Baseline diagnostic workup for Cushing’s syndrome in the patient.

-	**Value**	**Response or Normal Range**
**UFC**	2133 nmol/24 h 1215 nmol/24 h	< 694 nmol/24 h
**LNSC**	9.1 nmol/l	< 3 nmol/l
**Serum cortisol after 1 mg-DST**	239 nmol/l	< 50 nmol/l
**ACTH**	21 ng/l	4.7 – 48.8 ng/l
**ACTH after hCRH**	23 → 94 ng/l	+ 308%
**Cortisol after hCRH**	429 → 974 nmol/l	+ 127%
**ACTH after DDAVP**	17 → 24 ng/l	+ 41%
**Cortisol after DDAVP**	498 → 797 nmol/l	+ 60%
**Serum cortisol after HDDST**	477 → 96 nmol/l	- 80%
**ACTH ** **BIPSS – basal ratio**	Right 41.13 Left 28.89	> 2 indicating CD
**ACTH** ** BIPSS – hCRH stimulated ratio**	Right 69.95 Left 36.2	> 3 indicating CD

**Abbreviations:** UFC, urinary free cortisol; LNSC, late-night salivary cortisol; DST, dexamethasone suppression test; ACTH, adrenocorticotropic hormone; hCRH, human corticotropin-releasing hormone; DDAVP, desmopressin; HDDST, high-dose dexamethasone suppression test; BIPSS, bilateral inferior petrous sinus sampling; CD, Cushing’s disease.

**Table 2:** Hormonal values at the last follow-up assessed 24 months after pasireotide withdrawal.

-		**2 Months after Drug Withdrawal**	**Last Follow-up**	**Response or Normal Range**
**UFC**		111 nmol/24 h 93 nmol/24 h	142 nmol/24 h 149 nmol/24 h	< 168 nmol/24 h
**LNSC**		2.3 nmol/l 1.8 nmol/l	1.1 nmol/l 1.1 nmol/l	< 2.6 nmol/l
**1 mg-DST**	**Serum cortisol**	n.a.	95 nmol/l	< 50 nmol/l
	**Dex levels**	n.a.	1.8 nmol/l	> 4.5 nmol/l
**Liddle test**	**Serum cortisol**	n.a.	35 nmol/l	< 50 nmol/l
	**Dex levels**	n.a.	8.3 nmol/l	> 4.5 nmol/l
**ACTH after DDAVP**		n.a.	17.2 → 27 ng/l	+ 57%
**Cortisol after DDAVP**		n.a.	200 → 270 nmol/l	+ 35%

**Abbreviations:** UFC, urinary free cortisol; LNSC, late-night salivary cortisol; DST, dexamethasone suppression test; n.a., not available; ACTH, adrenocorticotropic hormone; DDAVP, desmopressin; Dex, dexamethasone.

**Table 3:** Previous reports of sustained spontaneous CD remission after steroidogenesis inhibitor treatment.

Author, Year	Drug	Treatment duration	Follow-up	PTA suspicion	Relapse
Dickstein *et al*., (1991) [13]	Metyrapone	48 months	48 months	Presumptive	No
Dickstein *et al*., (1991) [13]	Ketoconazole	9 months	24 months	Radiological	No*
Nestour *et al*., (1994) [15]	Ketoconazole and Mitotane	40 months	20 months	Radiological	No
Pignatta *et al*., (2004) [16]	Ketoconazole	13 months	24 months	Clinical	No
Popa Ilie *et al*., (2021) [5]	Metyrapone	9 months	12 months	Presumptive	No

**Abbreviation:** PTA, pituitary tumour apoplexy. **Note:** *27 months after remission, despite a lack of hypercortisolism relapse, a pituitary tumour reappeared, and ACTH staining suggested the possibility of a phenotypic switch to a silent form [14].

**Table 4:** Previous reports regarding sustained remission after somatostatin analogue, cabergoline, or pegvisomant withdrawal in radiotherapy-naïve acromegalic patients.

Author, Year	N	Sustained Remission	Drugs	Follow-up
Avramidis *et al*., (2008) [31]	1	Case report	Octreotide LAR	6 years
Verhelst *et al*., (2008) [32]	2	Case reports	Cabergoline	2.5 years 5.5 years
Auriemma *et al*., (2010) [33]	1	Case report	Lanreotide LAR	24 months
Ramirez *et al*., (2012) [34]	12	5 [41.7%]	Octreotide LAR	12 months
Vilar *et al*., (2014) [35]	20	4 [20.0%]	Octreotide LAR	12-18 months
Hatipoglu *et al*., (2015) [36]	16	3 [18.8%]	Octreotide LAR Lanreotide LAR	12 months
Alvarez-Escola *et al*., (2016) [37]	1	Case report	Lanreotide LAR	3.5 years
Casagrande *et al*., (2017) [38]	58	3 [5.2%]	Octreotide LAR	18 months
Casagrande *et al*., (2017) [39]	12	1 [8.3%]	Cabergoline	108 weeks
	4	1 [25%]	Cabergoline + Octreotide LAR	
Puglisi *et al*., (2019) [40]	2	Case reports	Pegvisomant	2 years 5 years
Chiloiro *et al*., (2019) [23]	1	Case report	Pasireotide LAR	18 months
Sala *et al*., (2021)* [4, 41]	29	6 [20.7%]	Octreotide LAR Lanreotide LAR	120 months
Yu *et al*., (2022) [24]	1	Case report	Pasireotide LAR	21 months
Daly, (2022) [25]	1	Case report	Pasireotide LAR	5 years

**Abbreviations:** N, number of patients considered; LAR, long-acting release. **Note:** *Extension study of a previous work from the same group published in 2008 [41].

## Data Availability

All data underlying the results are available as part of the article and no additional source data are required.

## LIST OF ABBREVIATIONS

CD: Cushing’s Disease
LNSC: Late-night Salivary Cortisol
LAR: Long-acting Release
MRI: Magnetic Resonance Imaging
SSAs: Somatostatin Analogues
SSTR: Somatostatin Receptor
TSS: Trans Sphenoidal Surgery

## STANDARDS OF REPORTING

CARE guidelines have been followed in this study.
